# Lack of Association between the *MEF2A *Gene and Coronary Artery Disease in Iranian Families

**Published:** 2013-08

**Authors:** Kolsoum Inanloo Rahatloo, Saeid Davaran, Elahe Elahi

**Affiliations:** 1 School of Biology, University College of Science, Tehran, Iran; 2 Tehran University of Medical Sciences, Tehran, Iran

**Keywords:** Autosomal dominant, Coronary artery disease, *MEF2A*, Myocardial infarction

## Abstract

***Objective(s):*** Coronary artery disease (CAD) which may lead to myocardial infarction (MI) is a complex one. Great effort has been devoted to identification of genes that increase susceptibility to CAD or provide protection. A 21-bp deletion in the *MEF2A *gene, which encodes a member of the myocyte enhancer factor 2 family of transcription factors, has been reported in patients of a single pedigree that exhibited autosomal-dominant inheritance of CAD. Subsequent analysis of genetic variants within the gene in CAD and MI case-control settings produced inconsistent results. Here, we aimed at assessing the contribution of *MEF2A* to CAD in a cohort of Iranian CAD patients.

***Materials and Methods:*** Exon 11 of *MEF2A *wherein the above mentioned 21-bp deletion and a polyglutamine (CAG)_n_ polymorphism are positioned was sequenced by the dideoxy-nucleotide termination protocol. In 52 CAD patients from 12 families (3-7 affected members per family) and 76 Iranian control individuals. All exons of the gene were sequenced in 10 patients and 10 controls.

***Results:*** The 21-bp deletion was observed neither among the patients nor the control individuals. Four alleles of the polyglutamine (CAG)_n_ polymorphism were found, but there were no significant differences in allelic frequencies between patients and controls. Sequencing of all exons of MEF2A revealed the presence of 12 novel sequence variations in introns and flanking regions of *MEF2A* gene, not associated with disease status.

***Conclusion:*** Our data do not support a role for *MEF2A *in coronary artery disease in the Iranian patients studied

## Introduction

The evidence for heritability of coronary artery disease (CAD) is striking, with a positive family history being one of the most important risk factors for this complex trait (1). In recent years, there has been some progress in the identification of genes that are associated with susceptibility to the development of CAD. But, there has also been remarkable lack of replication among studies and difficulty in identifying genes with impressive linkage peaks (2). 

**Figure 1:**
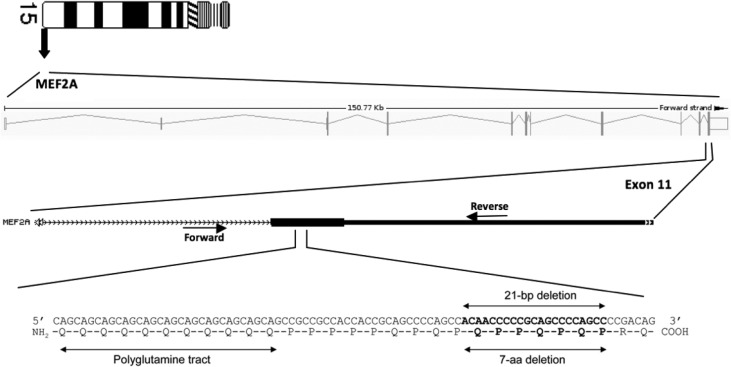
MEF2A gene. Schematic diagram of the human MEF2A gene is displayed in the upper panel. Exons and introns are drawn to scale. In the center panel, an enlargement of a portion of exon 11 is shown. Positions of primers are indicated by arrows. In the lower panel, an enlargement of exon 11 is shown. The corresponding nucleotide and amino acid sequences are displayed, and the polyglutamine tract and the 7 aa deletion (21-bp deletion) are indicated by arrows

Myocyte enhancer factor 2 (MEF2) constitutes a family of transcription factors composed of four members: MEF2A, MEF2B, MEF2C, and MEF2D. The MEF2 transcription factors bind to their cognate DNA sequence CAT(A/T)4TAG/A in the regulatory regions of several genes. The promoter regions of various genes expressed in the heart contain MEF2 binding sequences, and MEF2 could regulate inducible expression of some of these genes in muscle and endothelium of coronary arteries, among other cell types (3). The *MEF2A *gene, located at chromosome position 15q26.3, is composed of 11 exons spread over ~115 kb (Figure 1), and is expressed at high levels in skeletal, cardiac, smooth muscle, neuronal cells, and in the endothelium of coronary arteries (4, 5).

A deletion mutation in *MEF2A* was identified in a large pedigree with an autosomal dominant inheritance pattern of myocardial infarction (MI) and CAD (4). The *MEF2A* 21-bp deletion in exon 11 co-segregated with the presence or absence of CAD or MI in the family, and was not found in hundreds of controls without documented CAD by angiography (4). Furthermore, this deletion correlated with lack of nuclear translocation and a marked reduction of transcription activity. However, lack of replication has led to controversies regarding the role of MEF2A with respect to CAD (3, 6, 7). Weng *et al* identified the same *MEF2A* deletion in an individual who had a transient cerebrovascular attack (6, 7). Two of the proband’s siblings with the same deletion were reported as not having CAD or myocardial infarction, but none of these individuals had undergone coronary angiography and only one had stress testing. Beyond the 21-bp deletion, point mutations in exon 6 and 7 were reported to be associated with an increased risk of myocardial infarction, further implicating the association of *MEF2A* with coronary disease, and one of these point mutations, Pro297Leu, was independently replicated (3, 8). Although initial studies supported the involvement of *MEF2A* variants in the occurence of CAD/MI, these variants were later identified in healthy individuals, thus raising the possibility that they were rare DNA polymorphisms not directly related to the risk for CAD (6).

The role of *MEF2A *in the pathogenesis of cardiovascular diseases still remains uncertain. In fact, several case-control studies have been undertaken, but controversial results were found (3, 6, 9-12)*. *A large study by Lieb *et al* on more than 1700 patients with MI, 2 large control populations, and extended families with apparently Mendelian inheritance of the disease showed no evidence of an association between *MEF2A *and MI (13). Additionally, a meta-analysis provided no convincing evidence for the genetic involvement of *MEF2A* gene (CAG)_n_ polymorphism in CAD (14). The (CAG)_n_ tract encodes polyglutamine tandem repeats ((Q)_n_)in the protein product of the gene. 

 Here, we report results of screening the exon 11 21-bp deletion mutations and the CAG repeat polymorphism within the same exon of the *MEF2A* gene in 12 Iranian CAD pedigrees to assess whether an association exists between the *MEF2A* genetic variants and CAD in these pedigrees.

## Materials and Methods

***Ethics statement***

This research was performed in accordance with the Declaration of Helsinki and with approval of the ethics board of the University of Tehran. Participants or their guardians consented to participate after being informed of the nature of the research.

***Study population***

The study population comprised 12 families with 52 CAD patients and 76 healthy unrelated Iranian controls aged over 60 years. Coronary angiography revealed >50% stenosis in at least 1 major coronary artery in all the patients with CAD. Risk factors for CAD, such as hypertension, diabetes mellitus, hypercholesterolemia, and smoking were evaluated on the basis of the medical history, current medications, or the findings of examination during hospitalization. Obesity was defined as a body mass index (BMI) of 27 kg/m^2^ or more. The clinical features of the affected individuals were recorded. Controls were selected from Iranian peoples without any cardiovascular disease (41 women, 35 men; mean age = 56 years, SD = 10).

***Sequencing of MEF2A***

Blood samples (5 ml) were drawn and genomic DNA was extracted from peripheral blood leukocytes by standard phenol-chloroform extraction. 

Exon 11 of *MEF2A* was screened for mutations by direct sequencing in all 52 patients and 76 controls. The Sanger di-deoxy nucleotide termination protocol was used for sequencing. Exon 11 of *MEF2A* and its flanking intronic sequences in all the patients and controls were amplified by the polymerase chain reaction (PCR) and subsequently sequenced (Big Dye kit and the Prism 3700 sequencer; Applied Biosystems, Foster City, CA, USA). Primers used for PCR amplification and sequencing were designed by the Primer 3 software (http://frodo.wi.mit.edu/cgi bin/primer3/primer3) according to reference sequence (NM_005920.2) (Table 1). Sequences were analyzed through their comparison with the reference sequence (NT_037852.6) using Sequencher 4.8 software (Gene Codes, Ann Arbor, MI, USA). 

**Table 1:** Primer sequences used for amplification and sequencing of *Mef2A* exon 11

Primer name	Forward	Reverse
Mef11A	5'-GCCAAGCACTGAAGGAAACGAC-3'	5'-CATGCACCCCTTTGCAACAGAC-3'
Mef11A2	5'-CGTGGGTGACCTAAGGCTTCC-3'	5'-CATACACACTCACACCCACATAC-3'
Mef11B	5'-ACTAGCTTGCAGAAACCTAGAC-3'	5'-GAAACCCCTTTATACAATCCAC-3'
Mef11C	5'-ATTTATCACCTTTGATTAAGTACC-3'	5'-CTTGTCACAAACAGCAGATGAC-3'
Mef11D	5'-CATGAGCAAAATTCAAAGTCCTG-3'	5'-GTTGGAAACTGTACTTTAACCAG-3'
Mef11E	5'-AGGGGACGACGCTAATGGTG-3'	5'-TGCAGGTGAAAAAGGTCTTCGTG-3'
Mef11F	5'-GGGATCTTTTTTCCTTGACC-3'	5'-TAGTCTTTCTTTCTATGCAGG-3'

Finally, all exons of the *MEF2A *gene, including those that encode the 5´ and 3´untranslated regions, and flanking intronic boundaries were sequenced in two representative CAD patients from each of five extended families (n=10) and in 10 controls. The sequencing of all the exons was done using a Next Generation Sequencing protocol at Illumina (Illumina, San Diego, CA, USA).

***Statistical analysis***

The clinical characteristics of the continuous variables were expressed as mean ± SD, and were tested using 2-sample t-test. Allele frequencies in patients and controls were compared using χ^2^ test. All statistical analyses were done using SPSS statistical package (v.11.0).

## Results

***Clinical data***

Clinical data on classical CAD risk factors such as hypertension, hypercholesterolemia, diabetes mellitus, obesity, and smoking are presented in Table 2. The occurrence of all risk factors was higher among the patient cohort as compared to controls. 

***Genotyping of the 21-bp deletion, assessment of number of CAG repeats, and MEF2A sequence variations***

None of the 52 CAD affected individuals and none of the 76 control individuals carried the 21-bp deletion mutation. The allele distribution of the (CAG)_n_ polymorphism is shown in Table 3. The number of the CAG triplet repeats ranged from 9 to12 among both patients and controls. The frequencies of the alleles were similar in the patient and control groups, and the two groups showed no statistically significant difference in this regard (*P*=0.12). The majority of patients and the majority of controls had 9 or 11 repeats. 

Results of sequencing all exons of *MEF2A* by next generation sequencing in ten patients and ten controls, confirmed absence of the 21-bp deletion in these individuals. However, 31 sequence variations were found in *MEF2A* in the DNAs of the 20 individuals sequenced; 5 of these were found only in patients, 2 were found only in controls, and 24 were found in both groups (Table 4). Nineteen of the variations had been previously reported as polymorphisms, and 12 are novel variations. None of the novel variations were positioned within amino acid coding regions, and none were predicted to affect splicing. Five of the previously reported variations were within codons, and four of these affected synonymous changes (p.G443G, p.Q291Q, p.N289N and p.P472P). The only variation that caused an amino acid change (p.P421QQP) was observed in both patients and controls. None of the sequence variations within *MEF2A* were associated with CAD status. 

**Table 2:** Clinical features of the CAD patients and controls

	Controls	Patients	*P*
Number	75	52	
Age	56+10	53+6	0.015
Smoking,%	10.1	16.4	0.011
Obesity,%	18.2	23.1	0.015
Hypercholesterolemia,%	11.3	25.8	<0.001
Hypertriglyceridemia,%	13.4	26.2	0.015
Hypertension,%	23.8	39.7	<0.001
Diabetes Mellituse,%	5.1	32.4	<0.001

**Table 3:** Alleles of the (CAG)_n_ repeat in exon 11 (rs3138597) in Coronary Artery Disease patients and controls

Alleles with shown No. CAG repeats	9	10	11	12
Controls	64 (43 %)	14 (9.3%)	68 (45.1%)	4 (2.6%)
Patients	42 (40.1%)	10 (9.8%)	50 (48%)	2 (1.9%)

## Discussion

As already stated, results of investigations on association of *MEF2A *with CAD/MI have been inconclusive. In 2003, a 21-bp deletion in *MEF2A* was found to segregate with CAD in a large pedigree, and *MEF2A* was claimed as the first CAD causing gene that results in Mendelian inheritance of the disease (4). Among the 93 predicted genes within the identified linked locus, *MEF2A *was considered a plausible candidate because of its role in the development of heart muscle and its high level of expression in the endothelium of coronary arteries (4). Most importantly, its causal role was strengthened by in vitro functional studies showing that the 7 amino acid deletion caused by the 21-bp deletion and three missense mutations later found in patients, each either impaired the nuclear localization of the tanscription factor or decreased its transactivating activity (4, 5, 8). In this frame, Wang *et al* reported in the 2007 Scientific Sessions of the American Heart Association that *MEF2A *heterozygous knock-out mice (produced in an APOE/ background) showed markedly accelerated atherosclerosis. However, the association of *MEF2A *with CAD/MI has been challenged by subsequent investigations, because the originally described mutations in *MEF2A *were observed in unaffected individuals and did not segregate with the disease (6, 10).

**Table 4:** Genetic variants in* MEF2A* found by sequencing of 10 Iranian Coronary Artery Disease patients and 10 controls

SNP ID	Chromosome Position	Sequence around SNP	Type	Amino acid change	No. patients (n= 52)	No. controls (n=76)
New	100255013	CCCCC[C/A] CCACC	3´UTR	-	1	0
New	100255034	ATTAC [G/C] TTCCT	3´UTR	-	0	1
New	100092008	CTTTC[A/G]GACCT	Intronic	-	1	1
New	100215691	GTTTT[G/A]TAGGT	Intronic	-	1	2
New	100255016	CCCCC[A/C]CCCCC	3´UTR	-	1	1
New	100218867	CTGCT[A/G]AAAGA	Intronic	-	1	0
New	100230705	TTCCT[T/C]TGGAA	Intronic	-	1	1
New	100255021	ACCCC[C/A]CCCCC	Downstream	-	2	1
New	100092275	AAGGG[-/T]TTTGG	Intronic	-	1	1
New	100255017	CCCCA[CC/--]CCCCC	3´UTR	-	2	2
New	100255015	CCCCC[CA/--]CCCCC	3´UTR	-	2	3
New	100243506	AATAT[TTTG/----]TTTGT	Intronic	-	1	3
rs12902459	100255013	CCCCC[A/C]CCACC	3´UTR	-	2	0
rs58424802	100252738	GCAGC[------/AGCAGC] CGCCG	Exonic	P421QQP	1	1
rs141367967	100255016	CCCCA[--/CC]CCCCC	3´UTR	-	2	1
rs144314500	100255016	CCCCA[-/C]CCCCC	3´UTR	-	1	2
rs145618675	100253121	TGTGA[----/GTGT]GTGTG	3´UTR	-	1	1
rs28444186	100253128	TGTGT[A/G]TGTGT	3´UTR	-	1	0
rs325380	100256618	TTGTC[T/G]TCACC	3´UTR	-	1	2
rs325381	100255814	TCCCC[A/T]CTCTA	3´UTR	-	3	4
rs325382	100255104	CATAC[C/G]TATGT	3´UTR	-	1	3
rs325383	100255046	GAAAA[T/C]GTCAA	3´UTR	-	2	1
rs325399	100254444	CCTTA[T/C]AAAAT	3´UTR	-	2	2
rs325400	100252805	GAGCG[C/A]CCCAT	Exonic	G443G	1	1
rs325407	100246942	ATCCT[T/C]TGGGT	Exonic	Q291Q	1	2
rs325408	100246936	TGGGT[G/A]TTCTG	Exonic	N289N	1	0
rs34851361	100252892	TCTCC[A/G]ATTGT	Exonic	P472P	1	1
rs58267790	100255015	CCCCC[-/C]ACCCC	3´UTR	-	2	1
rs77710130	100256072	AAACT[C/T]CATCT	3´UTR	-	1	1
rs12902009	100106731	GCACC[C/G]CTTGG	5’UTR	-	0	1
rs897074	100254725	GGTGC[T/C]GGTCC	3´UTR	-	2	1

 Large-scale genome-wide CAD/MI association studies have now been performed to identify consistently replicated CAD/MI loci, significant signals in the 15q26 region wherein *MEF2A* is positioned, have not been observed (15-17). This is not surprising, because mutations in the Mendelian counterparts of common complex disease traits are often largely family specific and the role of the identified gene in pathogenesis of the disease at a population level are not easily detected (18). The role of *MEF2A *in MI was analyzed by Lieb *et al *in a large comprehensive analysis (13). The authors screened the entire gene in 23 patients with familial MI, genotyped the p.Pro279Leu variant in 533 patients with sporadic MI and 2076 controls, and analyzed the CAG repeat in 543 patients with sporadic MI and 1190 controls. Mutational screening failed to find the 21-bp deletion, and other pathogenic variants in *MEF2A *were not identified. Furthermore, analysis of single-nucleotide polymorphisms within the *MEF2A *gene in 753 patients and 1644 controls excluded their role in susceptibility for MI. However, an evaluation of the frequency of the 21-bp deletion in sporadic patients was lacking in this study (13).

Here, we screened 21-bp deletion in a total of 52 patients with CAD from 12 families and 75 healthy controls and sequenced all exons of the *MEF2A* gene in 10 CAD patients and 10 controls. We did not find the 21-bp deletion in any patient or control individual and did not observe a correlation between the CAG repeat number and disease status. Furthermore, we did not detect a correlation between disease status and any of the 31 variations found among the patient and control individuals of whom the gene was sequenced. Most of the variations were intronic or in regions that do not code amino acids. Taken together, our analyses indicate a lack of evidence for a significant association between CAD and *MEF2A *in the Iranian patients studied.

## Conclusion

Our analyses do not support the role of *MEF2A *in CAD, at least among the Iranian patients studied. The gene is unlikely to contribute to disease status in a notable fraction of Iranian CAD patients. 
